# Symptoms of depression as reported by Norwegian adolescents on the Short Mood and Feelings Questionnaire

**DOI:** 10.3389/fpsyg.2013.00613

**Published:** 2013-09-11

**Authors:** Astri J. Lundervold, Kyrre Breivik, Maj-Britt Posserud, Kjell Morten Stormark, Mari Hysing

**Affiliations:** ^1^Department of Biological and Medical Psychology, University of BergenBergen, Norway; ^2^Regional Centre for Child and Youth Mental Health and Child Welfare, Uni Health, Uni ResearchBergen, Norway; ^3^K. G. Jebsen Center for Research on Neuropsychiatric Disorders, University of BergenBergen, Norway; ^4^Department of Clinical Medicine, Haukeland University HospitalBergen, Norway

**Keywords:** depression, adolescent psychology, sex-differences, short mood and feelings questionnaire, factor analysis, statistical

## Abstract

The present study investigated sex-differences in reports of depressive symptoms on a Norwegian translation of the short version of the Mood and Feelings Questionnaire (SMFQ). The sample comprised 9702 Norwegian adolescents (born 1993–1995, 54.9% girls), mainly attending highschool. A set of statistical analyses were run to investigate the dimensionality of the SMFQ. Girls scored significantly higher than boys on the SMFQ and used the most severe response-category far more frequently. Overall, the statistical analyses supported the essential unidimensionality of SMFQ. However, the items with the highest loadings according to the bifactor analysis, reflecting problems related to tiredness, restlessness and concentration difficulties, indicated that some of the symptoms may both be independent of and part of the symptomatology of depression. Measurement invariance analysis showed that girls scored slightly higher on some items when taking the latent variable into account; girls had a lower threshold for reporting mood problems and problems related to tiredness than boys, who showed a marginally lower threshold for reporting that no-one loved them. However, the effect on the total SMFQ score was marginal, supporting the use of the Norwegian translation of SMFQ as a continuous variable in further studies of adolescents.

## Introduction

Psychiatric disorders are highly frequent in adolescence (Costello et al., 2011). Depression is probably the most frequent of them all (Kessler et al., 2005), with female gender as one of the main risk factors (Klein et al., 2013). The greatest increase in sex-difference occurs during adolescence (Hankin et al., 1998). At that age, population-based studies have shown that girls have a more than two-fold higher risk of depression than boys (Merikangas et al., 2010). This female:male ratio has been explained by sex-difference in pubertal development (Joinson et al., 2011, 2012; Black and Klein, 2012), but psychosocial (Nolen-Hoeksema, 1991; Copeland et al., 2009; Conley et al., 2012) and cognitive factors (Labelle et al., 2013) are probably as important.

Depressive symptomatology is associated with high rates of comorbid mental health problems (Angold et al., 1999a), social and academic dysfunction, suicide and substance abuse (Birmaher et al., 2007). Early identification and treatment may thus be crucial for present and future functioning. A main obstacle to early identification has been the lack of screening-instruments adapted to the child and adolescent language and daily life situations. The Mood and Feelings Questionnaire (MFQ) was developed as a response to that need (Angold et al., 1987). The questionnaire captures symptoms included in the DSM-IV criteria for major depressive disorders (Costello and Angold, 1988), and is an appropriate assessment tool in the age range 8–18 years. MFQ is validated in a large sample of both clinical and non-clinical subjects (Daviss et al., 2006) and has been translated and been part of population-based studies in Norway (Sund et al., 2003, 2011). A short version of MFQ (SMFQ) has also been developed as a promising option to validly assess core depressive symptoms in epidemiological studies (Angold et al., 1995). A strong unidimensionality and high internal consistency of SMFQ have been documented in a population-based study (Sharp et al., 2006).

The items included in SMFQ assess mood and anhedonia, tiredness, restlessness, concentration difficulties, and several aspects of negative self-evaluation. In their Item Response Theory (IRT) analysis, Sharp et al. (2006) showed that SMFQ items reflecting negative self-evaluation (referred to as cognitive by Sharp et al.) were characterized by high loadings on a unidimensional latent factor of depression. According to Nolen-Hoeksema (1991), different responses on such items may explain the sex differences seen in adolescents. Girls tend to ruminate more than boys, leading to a passive and repeated focus on ones depressive symptoms, causes and consequences (Watkins and Moulds, 2005). Rumination is shown to increase the vulnerability to develop depressive symptoms, and tends to increase the duration and severity of existing symptoms (Abela and Hankin, 2011). On the other hand, such thoughts may be easier to handle for girls than for boys, in that their social skills may help them to get support. In a population-based study of 13–17 years olds, Derdikman-Eiron et al. (2012) showed that symptoms of anxiety and depressions actually had more negative consequences for boys than for girls; when boys reported depressive symptoms, these symptoms had a stronger impact on their function. Finally, it is also a question as to whether a more affective response-style in girls than boys contributes to reports of more severe depressive symptoms in girls. This was indicated by a recent study of 8–17 years olds (van Beek et al., 2012), which showed that girls tend to report more severe problems on a questionnaire than expected from an evaluation based on a formal clinical assessment.

Assessment using questionnaires is challenged by the fact that depressive symptoms are very common, and that there is not a clear-cut distinction between case and noncase. This is especially true for emotional problems in adolescence, when feelings of misery and unhappiness are part of normal development. These mood related problems might be hard to differentiate from symptoms that are predictive of persistent mental health problems into adulthood. The importance of symptoms below the threshold of a diagnosis of depression in adolescents was illustrated in a study by Fergusson et al. (2005). In a 25-year longitudinal study of a birth cohort including 1265 children, they showed that symptoms of depression at a subclinical level at age 17–18 represented a highly relevant risk factor of depressive disorder and suicidal behavior later in life. The importance of subclinical symptoms of depression was also illustrated by results in a study of Norwegian families where an adolescent had committed suicide (Freuchen et al., 2012). Retrospectively, parents frequently had noticed symptoms of depressed mood before the fatal behavior, but at a level that did not elicit enough worry to engage professional help. These studies emphasize the importance of evaluating reports on questionnaires as a continuous rather than categorical variable, and to reveal symptoms that are predictive of persistent psychiatric problems and self-harm.

The aim of the present study was to investigate sex-difference in SMFQ reports in a large population-based sample of adolescents. Although the unidimensionality of SMFQ is known from an earlier study (Sharp et al., 2006), we found it necessary to analyse the factor structure of the Norwegian translation as part of the present study, separately for boys and for girls, with follow-up of differences with measurement invariance analyses. Revealing unidimensionality would enable using the total SMFQ score as a continuous variable representing severity of depressive symptoms. From earlier studies we expected to find that girls would report depressive symptoms more frequently and at a more severe level than boys.

## Methods

The present study included data from the ung@hordaland-survey of adolescents in the county of Hordaland, Western Norway, where all adolescents born between 1993 and 1995 living in the county and all students attending highschool during the spring 2012 were invited to participate. The adolescents received information via e-mail, and one school hour was allocated for them to complete the questionnaire. Adolescents not in school received information by postal mail to their home addresses. The questionnaire was web-based and covered a broad range of mental health- and demographic background variables. The Regional Center for Child and Youth Mental Health and Child Welfare, Uni Research, collaborated with Hordaland County Council to conduct the study. The study was approved by the Regional Committee for Medical and Health Research Ethics in Western Norway. The current study is based on the first version of quality-assured data files released in May 2012, and included the adolescent's self-reports on the SMFQ.

### Sample

Of the included adolescents (born 1993–1995, *N* = 19, 121), 10,220 completed or filled in parts of the questionnaire, yielding a participation rate of 53%. A total of 518 adolescents were excluded due to missing reports on some of the SMFQ items, resulting in a total sample of 9702 adolescents included in the present study. Information about sex and year of birth were based on the personal identity number in the Norwegian National Population Register.

### Short mood and feelings questionnaire

In the original version of the MFQ, the respondents are asked to rate 33 items on a 3-point scale, indicating how much they have felt or acted that way during the past 2 weeks (Angold et al., 1987). In the present study we included the official Norwegian version of the short version (SMFQ) (Angold et al., 1995), with 13 items focusing on affective and cognitive symptoms. The wordings of the response-categories in the Norwegian translation equals the original categories of *Not true*, *Sometimes true*, and *True*.

### Statistical analyses

SPSS version 18 was applied for descriptive statistics (SPSS I, 1999). The remaining analyses were conducted using Mplus version 7.0 (Muthen and Muthen, 2012). Missing data were dealt with using pair-wise deletion, which is Mplus default when analysing categorical outcome variables with the robust-weighted least square estimator (WLSMV). Confirmatory Factor Analyses (CFAs) were used on reports from girls and boys separately. The WLSMV was used in the CFAs due to highly skewed categorical data (ordinal data with three options). WLMSV uses polychoric correlations for estimation and seems relatively robust to violations of normality (Flora and Curran, 2004; Brown, 2006). Bentlers comparative fit index (CFI) (Bentler, 1990), TuckerÐLewis Index (TLI) (Tucker and Lewis, 1973) and the root-mean-square error of approximation (RMSEA) (Steiger and Lind, 1980) were used for evaluation of model fit. The cut-off values to determine acceptable fit followed the suggestions given by Yu (2002): CFI 0.96, TLI 0.95 and RMSEA 0.05. An adjusted chi-square test (DIFFTEST) (Muthen and Muthen, 2012) will be reported, as it is appropriate when comparing difference in fit between nested models. Explorative bifactor analyses were conducted when the hypothesized unidimensional model did not fit the data.

Measurement invariance between boys and girls was tested by using a top down strategy (Muthen and Muthen, 2012), where the fit of a model of which the loadings and thresholds were held equal between the sexes was compared to a model of which the same parameters (except for the identification item) were free to vary. The model was assumed non-invariant if the adjusted chi square was significant and the change in CFI was <= −0.002 (Meade et al., 2008). The loadings and threshold (in tandem) of potentially problematic items (based on having a modification index >10) were then sequently set to be free between the sexes.

## Results

### Demographics

The mean age of the sample was 17 years (*SD* = 0.87) and 53.5% of the respondents were girls (*n* = 5188). The majority (98%) were highschool students. The proportion of immigrants in the present sample, defined as having both parents born outside Norway, was 5.6% (*n* = 545).

### Factor models for depressive symptoms in boys and girls

The 1 factor (unidimensional) confirmatory model had only a modest fit to data for both boys and girls in the present sample, with an adequate fit for the CFI and TLI (girls: CFI = 0.96, TLI = 0.96; boys: CFI = 0.97, TLI = 0.97), but a somewhat high RMSEA (0.09 for girls and 0.07 for boys). Inspection of the modification indexes suggested that the misfit could partly be due to local dependencies between items 3, 4, 7, items 1, 2, and items 8, 9. Explorative factor analyses [estimator = geomin rotated solution (oblique)] on the polychorical correlation matrix were therefore conducted. A similar three factor solution with good fit and theoretical sense was found for both sexes (negative self-evaluations: item 5, 8, 9, 10, 11, 12, 13; negative mood: item 1, 2, and 6; and a factor assessing restlessness, tiredness and concentration problems: item 3, 4, and 7). The results from the explorative factor analyses suggested, however, that the data were characterized by one dominant factor as the ratio between the first and second eigenvalue was large for both girls (7.87 vs. 1.15, 61% of the variance explained by the first factor) and boys (8.96 vs. 1.01, 67% of the variance explained by the first factor).

Explorative bifactor analyses (Bi-cf-quartimax (orthogonal) rotation) with a four factor solution were therefore conducted. The fit of this model was excellent for both sexes (RMSEA < 0.04 and CFI > 0.99). All items except item 4 (restlessness) had a rather strong loading (>0.60) on the general factor (Tables 1, 2). The loadings on the domain specific factors correspond to a certain extent with what was discovered in the EFA analyses.

**Table 1:** **The factor structure of the Norwegian translation of SMFQ: girls**.

**Item**	**Title**	**Uni**	**Gen**	**Sp1**	**Sp2**	**Sp3**
Item 1	I felt miserable or unhappy	0.81^*^	0.78^*^	0.56^*^	0.01	−0.02^*^
Item 2	I didn't enjoy anything	0.70^*^	0.69^*^	0.22^*^	0.14^*^	−0.09^*^
Item 3	I felt so tired I just sat around and did nothing	0.62^*^	0.62^*^	0.06^*^	0.43^*^	−0.13^*^
Item 4	I was restless	0.38^*^	0.37^*^	−0.05^*^	0.45^*^	0.01^*^
Item 5	I felt I was no good any more	0.86^*^	0.86^*^	0.04	−0.07^*^	0.10^*^
Item 6	I cried a lot	0.74^*^	0.70^*^	0.36^*^	0.04^*^	0.09^*^
Item 7	I found it hard to think properly or concentrate	0.67^*^	0.66^*^	0.04^*^	0.41^*^	0.08^*^
Item 8	I hated myself	0.89^*^	0.85^*^	0.02	−0.06^*^	0.34^*^
Item 9	I was a bad person	0.86^*^	0.81^*^	−0.01	0.01	0.41^*^
Item 10	I felt lonely	0.78^*^	0.80^*^	0.05^*^	−0.12^*^	−0.04
Item 11	I thought nobody really loved me	0.86^*^	0.89^*^	−0.14^*^	−0.25^*^	−0.01
Item 12	I though I could never be as good as other kids	0.82^*^	0.82^*^	−0.17^*^	−0.10^*^	0.18^*^
Item 13	I did everything wrong	0.85^*^	0.83^*^	−0.07^*^	0.02	0.22^*^

*Uni, Unidimensional model from confirmatory factor analysis; Gen, the general factor from the bifactor analysis (multi group); Sp1, the first domain specific factor; Sp2, the second domain specific factor; Sp3, the third domain specific factor*.

* *p < 0.05. Bold values, see text*.

**Table 2:** **The factor structure of the Norwegian translation of SMFQ: boys**.

**Item**	**Title**	**Uni**	**Gen**	**Sp1**	**Sp2**	**Sp3**
Item 1	I felt miserable or unhappy	0.82^*^	0.82^*^	0.40^*^	−0.04^*^	−0.04^*^
Item 2	I didn't enjoy anything	0.74^*^	0.73^*^	0.29^*^	0.13^*^	−0.02
Item 3	I felt so tired I just sat around and did nothing	0.66^*^	0.63^*^	0.09^*^	0.45^*^	−0.08^*^
Item 4	I was restless	0.48^*^	0.44^*^	−0.07^*^	0.43^*^	0.07^*^
Item 5	I felt I was no good any more	0.88^*^	0.89^*^	−0.03	−0.02	0.06^*^
Item 6	I cried a lot	0.82^*^	0.81^*^	0.21^*^	−0.02	0.15^*^
Item 7	I found it hard to think properly or concentrate	0.72^*^	0.70^*^	−0.04	0.43^*^	0.02^*^
Item 8	I hated myself	0.92^*^	0.89^*^	−0.01	−0.04	0.28^*^
Item 9	I was a bad person	0.89^*^	0.86^*^	−0.03	−0.01	0.36^*^
Item 10	I felt lonely	0.83^*^	0.86^*^	0.02	−0.12^*^	−0.18^*^
Item 11	I thought nobody really loved me	0.91^*^	0.92^*^	−0.09^*^	−0.15^*^	0.01
Item 12	I though I could never be as good as other kids	0.87^*^	0.88^*^	−0.21^*^	−0.02	0.04
Item 13	I did everything wrong	0.88^*^	0.87^*^	−0.10^*^	0.04	0.21^*^

*Uni, Unidimensional model from confirmatory factor analysis; Gen, the general factor from the bifactor analysis (multi group); Sp1, the first domain specific factor; Sp2, the second domain specific factor; Sp3, the third domain specific factor*.

* *p < 0.05. Bold values, see text*.

Although the results supported a dominant factor, the multi-group analyses Muthen and Muthen (2012) on the 1 factor solution suggested that some of the items function differently for the two sexes. The fully constrained model, where the loadings and thresholds were set to equal between the two sexes, had a somewhat poorer fit (Δx2 = 552.28, df = 24, *p* < 0.001, ΔCFI = −0.002) compared to the least restrictive model where the same (except for the identification item) parameters were set free. Gradually releasing the equality constraints (based on the modification indexes) suggested that five of the items functioned somewhat differently between the sexes. Due to similar item loadings between the two sexes, they were constrained to be equal, resulting in a final partial invariance model which still had a somewhat poorer fit than the least unrestricted model (Δx2 = 81.44, *df* = 19, *p* < 0.001).

The CFI index was in fact larger (ΔCFI = 0.009) for the partial invariance model, which supports its greater parsimony compared to the least restrictive model. Given the same level of depression, girls had a lower threshold for reporting that they felt miserable or unhappy (1), to feel so tired they just sat around doing nothing (3) and to cry a lot (6) than boys. Boys had a marginally lower threshold to report that they believed no one loved them (11) than girls with a similar depression trait value. Taking the different item functions (DIF) into account had a minimal impact on the estimated mean sex-difference, as girls had 1.05 Standard deviation units higher score based on latent mean depression score than boys in the final partial measurement model [Cohens d (Cohen, 1988)Δ 0.47 latent mean, variance girls 0.622, boys 0.708], compared to 0.98 Standard deviation units (latent mean Δ 0.68 variance girls = 0.653, boys = 0.943) when the fully constrained model was used.

### Total SMFQ score

**Figure 1:**
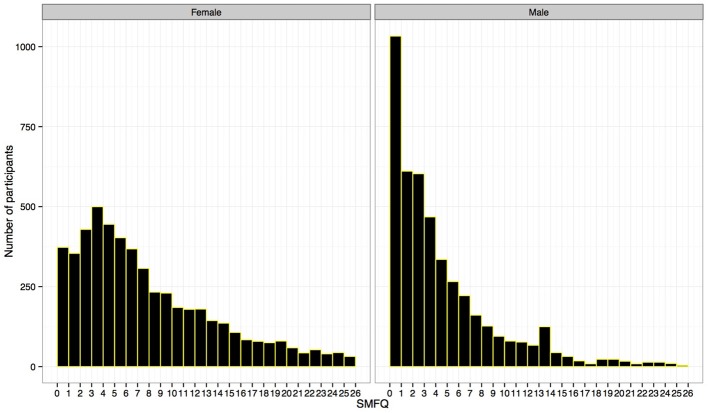
**Distribution of scores on the Short Mood and Feelings Questionnaire (SMFQ), given in separate plots for females (to the left) and males (to the right)**.

The high factor loadings on the general factor allowed using the total SMFQ score as an indicator of severity. Girls obtained a statistically significant higher mean score (7.4 (6.1)) than boys (4.1 (4.9), *t* = 28.8, *p* < 0.001), with a medium to high effect-size (*d* = 0.60). Figure 1, showing the distribution of the scores within the two sex-groups, revealed that a high number of boys reported no problems on all items (i.e., a score = 0). Although the frequency was lower than for girls on most other scores, the boys were distributed throughout the most rightward length.

### Sex differences in scores on the SMFQ items

The percentage of responses within the three response-categories are given in Table 3. A high percentage of female adolescents answered *True* on one or more items, ranging from 5.6% up to 25.9%, with the highest reports on items assessing mood problems (1), tiredness (3), poor concentration (7), loneliness (10) and feeling of being no good (5). In general, girls used the response-category *True* significantly more frequently than boys on all items (*p* < 0.001). The female:male ratios (OR) of using the most extreme response options were high, ranging from about two-fold up to a more than five-fold increase (cried a lot).

**Table 3:** **Percentage of girls and boys with a given response on the SMFQ items**.

	**Not true**		**Sometimes true**		**True**		
	**Girls**	**Boys**	**Girls**	**Boys**	**Girls**	**Boys**	**OR**
Item 1	34.4	67.1	45.1	26.0	20.5	7.0^**^	2.9
Item 2	55.4	70.2	32.5	23.6	12.1	6.2^**^	1.9
Item 3	27.5	49.4	46.5	38.4	25.9	12.2^**^	2.1
Item 4	44.8	55.0	41.1	34.7	14.1	10.2^**^	1.4
Item 5	57.7	77.5	28.2	16.7	14.0	5.8^**^	2.4
Item 6	64.2	91.9	24.7	6.1	11.0	2.0^**^	5.5
Item 7	36.5	54.8	43.7	35.9	19.8	9.3^**^	2.1
Item 8	75.1	87.5	16.7	9.4	8.2	3.2^**^	2.6
Item 9	68.9	83.0	22.7	13.4	8.3	3.6^**^	2.3
Item 10	54.4	71.6	30.5	21.3	15.1	7.1^**^	2.1
Item 11	77.6	86.8	15.1	9.5	7.3	3.7^**^	2.0
Item 12	65.2	81.4	24.3	13.8	10.5	4.8^**^	2.2
Item 13	72.3	84.3	20.1	12.1	7.6	3.6^**^	2.1

** *p < 0.001: report of True from girls on an item compared to this report from boys. OR, Odds-ratios for girls to report True compared to boys*.

## Discussion

The present study showed that the Norwegian translation of SMFQ is essentially unidimensional, with one dominant depression factor explaining much of the item responses. By this, the use of the sum score of SMFQ as a continuous measure of severity was supported. There was a high rate of depressive symptoms in this population-based sample of 16–19 years old adolescents. The frequency of girls using the most severe response category (i.e., *True*) to describe their problems was higher than for boys. Among those reporting symptoms, a high proportion of the boys reported *Sometimes true* on the items, with the highest frequency on items reflecting tiredness, restlessness and concentration problems.

The higher scores on items assessing depressive symptoms in girls than in boys confirmed results from several previous studies (Angold et al., 2002; Merikangas et al., 2010; Joinson et al., 2011), with the present study showing a difference in the magnitude of medium to high effect size. A more severe depression in girls than boys was also confirmed by the multi-group analysis. The importance of being aware of depressive symptoms in girls has been emphasized by studies showing that such symptoms place them at risk for future social and academic problems (Birmaher et al., 2007), as well as related problems such as suicidal thoughts/behavior (Fergusson et al., 2005) and drug problems (Fombonne et al., 2001). Findings in a recent study by Monshouwer et al. (2012) showed the importance of detecting depressive symptoms in girls at an early adolescent age: onset of depression when the child was 15 years or younger was predictive of more long-lasting and severe future episodes of depression than for adolescents with a later onset.

Although not as frequent as in girls, a high number of boys did report problems. Problems relating to mood, anhedonia, hypo-activity, poor concentration and feeling of loneliness were reported as *Sometimes true* or *True* by between 20 and 30% of the boys. The importance of being aware of and help boys to cope with depressive symptoms was emphasized by a longitudinal population-based study of children from 13 to 19 years (Derdikman-Eiron et al., 2012), showing that symptoms of depression during this age period were related to even larger functional impairment for boys than for girls; boys showed more academic, behavioral and social problems than girls after remission. If we assume that at least some of the boys are impaired even though they use a less severe response category than girls, it may be crucial to give such responses attention. However, further studies validating reports on SMFQ against conventional clinical diagnostic interviews are needed to investigate this assumption.

The factor analysis of the Norwegian translation of SMFQ is one of the main contributions of the present study. Overall, the results confirmed the essential unidimensionality of SMFQ described by Sharp et al. (2006), although they included a much younger sample (7–11 years) and showed a similar factor structure for girls and boys. These findings of a strong depression factor gave solid arguments for comparing adolescent boys and girls on a sum-scale of severity. At the same time, the sex difference was noteworthy higher if the degree of depression in girls and boys was compared by the use of the latent depression variable (Cohens *d* = 0.98) instead of the sumscore (Cohens *d* = 0.60). Future sex-comparisons on the SMFQ should therefore be conducted on the latent variable as this takes into account error variance and the fact that the items are at an ordinal level.

In their IRT analysis, Sharp et al. (2006) showed that some of the items that we have referred to as part of a negative self-evaluation factor (8: hated self, 9: bad person, 13: did everything wrong) showed a high discriminating power on all levels of the SMFQ. These items were allocated to one of the three bifactors in the present study. One should, however, be careful in treating these domain specific factors (related to negative mood, negative self-evaluation and psychomotor agitation/somatic) as subscales. The results from the bifactor analysis suggest that such subscales would be rather useless for measuring domain specific content due to low reliability. Items belonging to these domain specific factors most often had a much higher loading on the general factor. Such subscales would therefore to a large extent reflect the general factor instead of the domain specific area of which it would be labeled.

Although a follow-up analysis showed that they loaded strongly on a general factor, an interesting sex-difference emerged. Given the same level of depression, girls had a lower threshold for reporting crying and feeling miserable and unhappy and being tired than boys. The sex difference related to crying has been shown in many previous studies on depression scales [e.g., (Teresi et al., 2009)] and probably reflects different gender norms of how acceptable it is to cry when feeling sad. It may also indicate that girls are somewhat more aware of negative feelings when depressed, as this could explain the tie between the reports of crying and feeling miserable and unhappy. Interestingly, boys were somewhat more likely to report that no one loved them than girls at a similar level of depression. We are hesitant to interpret this sex-difference until it is replicated as it was marginal. However, the results showed only a minor impact on the scale level. This supports that comparisons of depression can be made using the sum-score of SMFQ despite the fact that some items functioned differently for boys and girls.

Sharp et al. (2006) found the items related to tiredness (3), restlessness (4) and concentration (7) to have a very weak discriminative power in the severe end of the SMFQ. These three items were also found to have the most ambiguous loadings in the bifactor model of the present study, and it is thus tempting to suggest that this bifactor is related to sleep and/or concentration difficulties that adolescents may suffer from both independently and in conjunction with depressive symptomatology. First of all, restlessness and tiredness may be related to changes in the sleep-wake-pattern during adolescence, due to physiological and psychological factors (Gradisar et al., 2011). Problems related to concentration, with huge impact on adolescents attending highschool education, may be closely related to brain development (Buitelaar, 2012) as well as a range of contextual factors. Further studies are called for, both given the established link between depression and insomnia (Roane and Taylor, 2008; Sivertsen et al., 2012; Danielsson et al., 2013) and between depression and cognitive problems (Austin et al., 2001; Ardal et al., 2012; Hammar and Ardal, 2012).

### Strengths and limitations

The large sample-size and the statistical analysis of the Norwegian translation of the SMFQ are main strengths of the present study. It is, however, important to be aware that we used a short version of MFQ, which does not include important symptoms of depression like suicidality/self-harm, rumination and biological features like eating little/much, sleeping little/much. In addition to the danger of missing some important aspects of depression, our results did also indicate that several symptoms are probably shared by other disorders, indicating that SMFQ may also be overinclusive.

The lack of including information regarding co-occurring problems is probably a main limitation of the present study, in that co-existence of mental health problems tends to be the rule rather than the exception (Gillberg, 2010). Studies have shown that between 40 and 90 percent of adolescents with depressive disorders have other psychiatric disorders, of whom about half have two or more diagnoses (Birmaher et al., 2007). In adolescents, comorbidity with anxiety as well as ADHD and other neuro-developmental disorders are frequent and have a substantial influence on persistence and future function (Angold et al., 1999b). These co-existing problems or disorders could account for some of the gender differences, as well as some of the weaker factor loadings. Furthermore, it was beyond the scope of the present study to investigate if there are differences in functional outcomes for girls and boys on factors such as school functioning and leisure time activities. Finally, the cross-sectional design of the present study does not allow for investigation of developmental pathways; questions related to the development of gender differences in depressive symptoms from childhood to adolescence await longitudinal studies.

## Conclusions

The present study confirmed the essential unidimensionality of the SMFQ in a Norwegian population-based sample of adolescents attending highschool. Adolescent girls reported depressive symptoms more frequently and at a more severe level than adolescent boys. Even though some of the SMFQ items seem to function somewhat different for boys and girls, these differences did not lead to biased results.

Reports on some of the items, e.g., those related to restlessness, tiredness and concentration problems, seemed to be both part of and independent of depressive symptomatology. The high number of adolescents reporting problems reflecting depressive symptoms is alarming from a public health perspective. Although only a fraction of the adolescents with a high score on the SMFQ will show a severity and a functional impairment confirming a diagnosis of depression, it is reasonable to assume that most of them have problems related to everyday functioning. The findings in the present study thus call for further studies, both to investigate co-existing problems, and risk and resilience factors associated with developmental pathways of depressive symptoms.

### Conflict of interest statement

The authors declare that the research was conducted in the absence of any commercial or financial relationships that could be construed as a potential conflict of interest.
